# Diagnostic value of [18F]FDG-PET/CT in polymyalgia rheumatica: a systematic review and meta-analysis

**DOI:** 10.1007/s00259-020-05162-6

**Published:** 2020-12-28

**Authors:** K. S. M. van der Geest, G. Treglia, A. W. J. M. Glaudemans, E. Brouwer, F. Jamar, R. H. J. A. Slart, O. Gheysens

**Affiliations:** 1 Department of Rheumatology and Clinical Immunology, University of Groningen, University Medical Center Groningen, Hanzeplein 1, 9700RB Groningen, the Netherlands; 2 Clinic of Nuclear Medicine and PET/CT Center, Imaging Institute of Southern Switzerland, Ente Ospedaliero Cantonale, Bellinzona, Switzerland; 3 Clinic of Nuclear Medicine and PET/CT Center, Imaging Institute of Southern Switzerland, Ente Ospedaliero Cantonale, Lugano, Switzerland; 4 Department of Nuclear Medicine and Molecular Imaging, Lausanne University Hospital, University of Lausanne, Lausanne, Switzerland; 5 Health Technology Assessment Unit, Academic Education, Research and Innovation Area, Ente Ospedaliero Cantonale, Bellinzona, Switzerland; 6 Department of Nuclear Medicine and Molecular Imaging, University of Groningen, University Medical Center Groningen, Groningen, The Netherlands; 7 Department of Nuclear Medicine, Cliniques Universitaires Saint-Luc and Institute of Clinical and Experimental Research (IREC), Université Catholique de Louvain (UCLouvain), Brussels, Belgium; 8 Department of Biomedical Photonic Imaging, Faculty of Science and Technology, University of Twente, Enschede, The Netherlands

**Keywords:** Polymyalgia rheumatica, Positron emission tomography/computed tomography, Fluorodeoxyglucose F18, Meta-analysis, Review

## Abstract

**Purpose:**

Polymyalgia rheumatica (PMR) can be difficult to diagnose. Whole-body [18F]FDG-PET/CT allows for a comprehensive evaluation of all relevant articular and extra-articular structures affected by PMR. We aimed to summarize current evidence on the diagnostic value of [18F]FDG-PET/CT for a diagnosis of PMR.

**Methods:**

PubMed/MEDLINE and the Cochrane Library database were searched from inception through May 31, 2020. Studies containing patients with PMR who underwent [18F]FDG-PET/CT were included. Screening and full-text review were performed by 3 investigators and data extraction by 2 investigators. Risk of bias was examined with the QUADAS-2 tool. Diagnostic test meta-analysis was performed with a bivariate model.

**Results:**

Twenty studies were included in the systematic review, of which 9 studies (*n* = 636 patients) were eligible for meta-analysis. [18F]FDG positivity at the following sites was associated with a diagnosis of PMR: interspinous bursae (positive likelihood ratio (LR+) 4.00; 95% CI 1.84–8.71), hips (LR+ 2.91; 95% CI 2.09–4.05), ischial tuberosities (LR+ 2.86; 95% CI 1.91–4.28), shoulders (LR+ 2.57; 95% CI 1.24–5.32) and sternoclavicular joints (LR+ 2.31; 95% CI 1.33–4.02). Negative likelihood ratios (LR−) for these sites, as well as the greater trochanters, were all less than 0.50. Composite [18F]FDG-PET/CT scores, as reported in 3 studies, provided a pooled LR+ of 3.91 (95% CI 2.42–6.32) and LR− of 0.19 (95% CI 0.10–0.36). Moderate to high heterogeneity was observed across the studies, mainly due to differences in patient selection, scanning procedures and/or interpretation criteria.

**Conclusion:**

Significant [18F]FDG uptake at a combination of anatomic sites is informative for a diagnosis of PMR. [18F]FDG-PET/CT might be an important diagnostic tool in patients with suspected PMR. This study also highlights the need for adherence to published procedural recommendations and standardized interpretation criteria for the use of [18F]FDG-PET/CT in PMR.

**Supplementary Information:**

The online version contains supplementary material available at 10.1007/s00259-020-05162-6.

## Introduction

Polymyalgia rheumatica (PMR) is the most common rheumatic inflammatory disease above the age of 50. It is characterized by inflammation of articular and peri-articular structures causing debilitating pain and stiffness of the shoulders and hips [1, 2]. PMR is associated with large vessel inflammation, i.e. giant cell arteritis, in approximately 20% of patients [2]. Inflammatory markers, such as the erythrocyte sedimentation rate (ESR) and C-reactive protein (CRP) level, are usually elevated in patients with PMR [3]. Several classification criteria have been proposed for PMR but these are not intended for diagnostic use [2]. There are no disease-specific symptoms or laboratory markers for PMR. The discrimination between PMR and its mimicking conditions can be very challenging. Since the treatment differs, the presence of other rheumatic diseases (e.g. late-onset rheumatoid arthritis, late-onset spondyloarthritis, osteoarthritis) as well as para-infectious myalgia and neoplastic diseases should be ruled out [4].

Various imaging modalities have been introduced in the diagnostic work-up of suspected PMR. Ultrasonography and magnetic resonance imaging (MRI) may reveal subacromial-subdeltoid bursitis, biceps tenosynovitis, glenohumeral synovitis, coxofemoral synovitis and/or trochanteric bursitis [2, 5–9]. These abnormalities are more accurately detected by MRI than ultrasonography [10]. MRI scans covering selected areas (e.g. shoulder and hip girdle) and also total body MRI may be helpful in the evaluation of PMR [7–10].

An emerging imaging tool for PMR might be 2-deoxy-2-[18F]fluoro-d-glucose ([18F]FDG) positron emission tomography combined with low-dose computed tomography ([18F]FDG-PET/CT). This imaging modality is well-established in oncology and has an expanding role in the assessment of inflammatory conditions [11, 12]. [18F]FDG enters activated immune cells and fibroblasts through the glucose transporter [13, 14]. Importantly, [18F]FDG-PET/CT allows for a comprehensive evaluation of all relevant articular and extra-articular structures in a patient with suspected PMR and may aid in the differentiation between PMR and other rheumatic inflammatory conditions [12, 15]. Furthermore, [18F]FDG-PET/CT allows ruling out concomitant large vessel vasculitis and other serious conditions [16]. In the current systematic review and meta-analysis, we aimed to summarize the growing evidence on the diagnostic value of [18F]FDG-PET/CT for a diagnosis of PMR.

## Methods

A predefined study protocol was established but not registered. This study is reported in agreement with the Preferred Reporting Items for a Systematic Review and Meta-Analysis (PRISMA) statement [17]. No ethical approval or informed consent was required.

### Search strategy

A comprehensive search of records through the PubMed/MEDLINE and Cochrane Library databases was carried out (date of last search: May 31, 2020). The following search algorithm was used: (A) ‘PET’ OR ‘positron emission tomography’ OR ‘FDG’ OR ‘fluorodeoxyglucose’ AND (B) ‘PMR’ OR ‘polymyalgia’. There were neither date limits nor language restrictions applied to the database search. In order to achieve a more comprehensive search, the references of the selected articles were screened manually.

### Study selection

Titles and abstracts of the records were independently screened by three reviewers (OG, GT and KSMG). Studies were selected for the systematic review according to predefined criteria. Inclusion criteria were original articles reporting [18F]FDG-PET/CT findings in patients with PMR. The reference standard for PMR could be classification criteria or a clinical diagnosis made by the treating physician. Exclusion criteria were as follows: (a) reviews, editorials, comments, study protocols; (b) case reports (less than 5 patients included); (c) articles outside the field of interest of this review (e.g. articles focused on [18F]FDG-PET without CT, articles including patients with giant cell arteritis rather than PMR); (d) articles not available in English. Subsequently, studies providing sufficient data on the diagnostic accuracy of [18F]FDG-PET/CT (i.e. the index text) for a diagnosis of PMR were included in the meta-analysis. Potential overlap of patients between studies from the same hospital was evaluated for studies in the meta-analysis. In case of possible overlap in patients, data was obtained from one study only and priority was given according to criteria in the following order: (1) a study with patients who were not (yet) treated with glucocorticoids, (2) a study with the largest number of patients, (3) a study reporting a clear definition of PET positivity, (4) a study including control subjects who were suspected of having PMR and (5) a study including control subjects with rheumatoid arthritis or another rheumatic inflammatory disease. Disagreements were solved through an online consensus meeting between the reviewers.

### Data extraction

Two reviewers (OG, GT) independently collected information about study characteristics (i.e. authors, year of publication, country, study design) and patient characteristics (i.e. patient population, criteria used for PMR diagnosis, age, sex ratio, number of PMR patients evaluated and [18F]FDG-PET/CT scans performed, immunosuppressive treatment, presence of a control group). Two independent reviewers (KSMG, RS) also collected data on technical details (i.e. [18F]FDG-PET/CT imaging modality, [18F]FDG injected activity, time interval between [18F]FDG injection and image acquisition, scan coverage, [18F]FDG-PET/CT image analysis and definition of positive findings) and any data on the per-patient diagnostic accuracy of [18F]FDG-PET/CT for PMR (i.e. true positive and true negative findings, false positive and false negative findings). Authors of studies were not contacted.

### Quality assessment

The quality of the studies included in the meta-analysis was assessed according to the revised ‘Quality Assessment of Diagnostic Accuracy Studies’ tool (QUADAS-2) [18]. The latter was used to assess the risk of bias for the following criteria: patient selection, index test, reference test and flow/timing whereas applicability concerns were assessed for patient selection, index test and reference test.

### Statistical analysis

A bivariate model analysis was performed to assess the summary estimates of sensitivity, specificity, diagnostic odds ratio (DOR), positive likelihood ratio (LR+) and negative likelihood ratio (LR−). Pooled data were given with 95% confidence intervals (95% CI) and displayed using forest plots and hierarchical summary receiver operating characteristics (HSROC) plots. Likelihood ratios of more than 2.00 or less than 0.50 with 95% CI not including 1.00 were considered statistically significant. The bivariate model analysis could not be used for findings reported by less than four studies. In that case, pooled estimates of the diagnostic parameters were determined with a univariate random effects model (DerSimonian-Laird method) and summary estimates were only shown if heterogeneity (*I*^2^) was < 75%. Bivariate model analysis and HSROC plots were performed with STATA version 15.1 (*metandi* command). Univariate models were evaluated with MetaDiSc version 1.4 and forest plots were constructed in Review Manager version 5.3. No sub-analyses were performed.

## Results

### Literature search

**Fig. 1:**
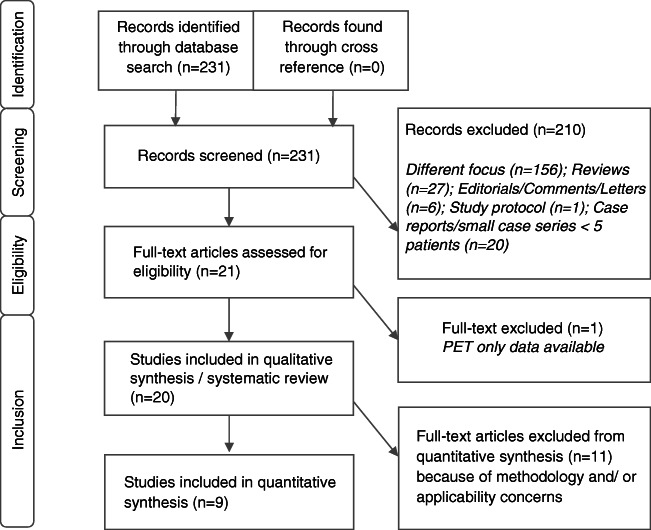
Study selection flowchart

A total of 231 records were identified through the comprehensive electronic database search (Fig. 1), with the oldest reference dating from May 1999 [19]. Two hundred ten records were excluded after title/abstract screening and 1 record after full-text evaluation [20]. Thus, 20 articles (*n* = 694 patients with PMR) were included in the qualitative analysis (systematic review) [21–40]. Subsequently, 11 of these studies were excluded from the meta-analysis due to lack of a control group (8 studies), inclusion of patients with giant cell arteritis without PMR (1 study), part of patients undergoing [18F]FDG-PET without CT (1 study) and one study reporting on muscle metabolic activity in patients with PMR rather than [18F]FDG uptake in typical joints, bursae and/or tendon entheses. Ultimately, 9 studies containing 636 patients (of which 253 patients had PMR) were eligible for the meta-analysis [21, 26, 30, 34–36, 38–40].

### Qualitative analysis (systematic review)

#### Basic study and patient characteristics

Table 1 summarizes the main characteristics of the 20 included studies. All selected articles have been published in the past decade. Eleven studies (55%) were performed in Europe, 7 studies (35%) in Japan and 2 studies (10%) in Australia. Thirteen studies (65%) had a retrospective study design, whilst 7 studies (35%) were performed prospectively. Thirteen studies (65%) included patients with PMR who underwent [18F]FDG-PET/CT at diagnosis before initiation of glucocorticoid therapy; in 7 studies (35%), at least part of patients had been treated with glucocorticoid treatment prior to or during the [18F]FDG-PET/CT. The reference standard for a diagnosis of PMR consisted of classification criteria in 17 studies (85%), i.e. the 2012 provisional ACR/EULAR classification criteria for PMR in 7 studies, Chuang’s criteria in 5 studies, Bird’s criteria in 2 studies, Healey’s criteria in 2 studies and a combination of the ACR/EULAR criteria and Bird’s criteria in 1 study [4, 41–43]. In 3 studies (15%), a clinical diagnosis of PMR was used as the reference standard. The included studies were heterogeneous concerning the sex and age of patients.

**Table 1:** [18F]FDG-PET/CT study and patient characteristics

Authors	Country	Study design	Patient population	Reference standard for PMR diagnosis	No. of PET/CT scans (PMR patients)	Median age (years), mean age (years)*	% male	Immunosuppressive treatment before PET/CT	Control group
Camellino et al. [21]	Italy	Prospective	PMR patients who had undergone ^18^F-FDG PET/CT at baseline	Bird’s criteria (retrospectively also fulfilling the ACR/EULAR criteria 2012)	65 (65)	73	32	No	Yes (OP and RA)
Charpentier et al. [22]	France	Retrospective	PMR patients who had undergone ^18^F-FDG PET/CT at baseline	ACR/EULAR criteria 2012	42 (42)	54* (young PMR) and 74* (elderly PMR)	65 (young PMR) and 29 (elderly group)	No	No
Cimmino et al. [23]	Italy	Prospective	PMR patients who had undergone ^18^F-FDG PET/CT at baseline or after therapy	Bird’s criteria	19 (19)	69*	44	In some cases	Yes (OP)
Devauchelle-Pensec et al. [24]	France	Prospective	PMR patients who had undergone ^18^F-FDG PET/CT at baseline and after therapy	Chuang’s criteria	60 (20)	67	65	Yes	No
Henckaerts et al. [25]	Belgium	Prospective	Suspected PMR patients who had undergone ^18^F-FDG PET/CT at baseline	Composite of clinical/biochemical/imaging results; confirmed by 6-month follow-up	not specified (67)	71	43	No	Yes (OD or ORD)
Horikoshi et al. [26]	Japan	Retrospective	PMR patients who had undergone ^18^F-FDG PET/CT at baseline	Composite of clinical/biochemical/imaging results	17 (17)	77 (75*)	53	No	Yes (OD or ORD)
Kaneko et al. [27]	Japan	Retrospective	PMR patients who had undergone ^18^F-FDG PET/CT at baseline	ACR/EULAR criteria 2012	20 (20)	73*	55	No	No
Lund-Petersen et al. [28]	Denmark	Retrospective	PMR patients who had undergone 18F-FDG PET/CT at baseline or after therapy	Unspecified clinical criteria	50 (50)	74	38	In some cases	No
Owen et al. [29]	Australia	Prospective	PMR patients who had undergone ^18^F-FDG PET/CT at baseline	ACR/EULAR criteria 2012	22 (22)	68*	59	No	No
Owen et al. [30]	Australia	Prospective	PMR patients who had undergone ^18^F-FDG PET/CT at baseline	ACR/EULAR criteria 2012	33 (33)	69*	55	No	Yes (OP or ORD)
Palard-Novello et al. [31]	France	Prospective	PMR patients who had undergone 18F-FDG PET/CT at baseline and after therapy	Chuang’s criteria	50 (18)	68*	67	Yes	No
Rehak et al. [33]	Czech Republic	Retrospective	PMR patients who had undergone ^18^F-FDG PET/CT at baseline	Healey’s criteria	35 (67)	70	43	No	No
Rehak et al. [32]	Czech Republic	Retrospective	PMR patients who had undergone 18F-FDG PET/CT at baseline and after therapy	ACR/EULAR criteria 2012	30 (15)	70	33	In some cases	No
Sondag et al. [34]	France	Retrospective	PMR patients who had undergone 18F-FDG PET/CT at baseline or after therapy	ACR/EULAR criteria 2012	50 (50)	69*	46	In some cases	Yes (OP)
Takahashi et al. [35]	Japan	Retrospective	PMR patients who had undergone ^18^F-FDG PET/CT at baseline	Chuang’s criteria (retrospectively also fulfilling Healey’s criteria)	27 (27)	78 (77*)	33	No	Yes (RA)
Wakura et al. [36]	Japan	Retrospective	PMR patients who had undergone ^18^F-FDG PET/CT at baseline	Healey’s criteria	15 (15)	72	33	No	Yes (RA)
Wendling et al. [37]	France	Retrospective	PMR patients who had undergone 18F-FDG PET/CT at baseline or after therapy	ACR/EULAR criteria 2012	101 (101)	69*	52	In some cases	Yes (OP)
Yamashita et al. [39]	Japan	Retrospective	PMR patients who had undergone ^18^F-FDG PET/CT at baseline	Chuang’s criteria (retrospectively also fulfilling Healey’s criteria)	14 (14)	73*	29	No	Yes (RA and ORD)
Yamashita et al. [38]	Japan	Retrospective	PMR patients who had undergone ^18^F-FDG PET/CT at baseline	Chuang’s criteria (retrospectively also fulfilling Healey’s criteria)	16 (16)	76*	25	No	Yes (SpA and RA)
Yuge et al. [40]	Japan	Retrospective	Suspected PMR patients who had undergone ^18^F-FDG PET/CT at baseline	ACR/EULAR criteria 2012 or Bird’s criteria	16 (16)	75*	6	No	Yes (OD or ORD)

*OP* oncological patients, *ORD* other rheumatic diseases, *RA* rheumatoid arthritis, *SpA* spondyloarthropathy

#### Technical aspects

The technical aspects of [18F]FDG-PET/CT in the 20 studies are summarized in Table 2. In 17 studies (85%), all patients underwent [18F]FDG-PET scanning with low-dose CT. The injected [18F]FDG activity was quite heterogeneous and included both weight-based and fixed activities. The [18F]FDG incubation time was approximately 60 min in all studies reporting this technical aspect. The vast majority of scans covered the skull (either from the vertex or skull base) to thigh region whilst some studies also included the knees. Reconstruction algorithms or adherence to EARL was not always specified. [18F]FDG-PET/CT image analysis was primarily performed by visual analysis (8 studies, 40%), semi-quantitative analysis using the maximum standardized uptake value (SUV_max_, 3 studies, 15%) or both of these methods (*n* = 9 studies, 45%). In two studies (10%), a target-to-liver ratio was used as well. The definition of a positive [18F]FDG uptake was different among the included studies, but the majority of studies used the liver as the reference organ. In 8 studies (40%), visual uptake equal or higher to the liver was considered positive whilst uptake higher than the liver (either visual or semi-quantitatively) was defined as positive in 5 studies (25%). Five studies (25%) reported a composite [18F]FDG-PET/CT score for PMR, but the anatomic regions included in the score differed per study (Supplementary Table 1).

**Table 2:** [18F]FDG-PET/CT characteristics in the studies

Study	Imaging modality	Injected activity	Interval [18F]FDG injection-image acquisition	Scan coverage	Image analysis	Definition of positive [18F]FDG-PET/CT finding
Camellino et al. [21]	PET/CT (low-dose CT)	4.8–5.2 MBq/kg	Unclear	Skull base to knee	Visual	Visual ≥ 2^b^
Charpentier et al. [22] ^d,g^	PET/CT (low-dose CT)	4.5 MBq/kg	60 min	Vertex to mid-thigh	Visual	Absent
Cimmino et al. [23] ^e^	PET/CT (low-dose CT)	4.8–5.2 MBq/kg	60–90 min	Skull base to knee	Visual	Uptake higher than the liver
Devauchelle et al. (2016)^d,g^	PET/CT (low-dose CT)	Unclear	Unclear	Unclear	Semi-quantitative (SUV_max_)	Absent
Henckaerts et al. [25] ^f^	PET or PET/CT (low-dose CT or diagnostic/contrast-enhanced CT)	4–5 MBq/kg	45–60 min	Whole body	Visual	1) Visual ≥ 2^b^ 2) Composite PET score cut-off
Horikoshi et al. [26]	PET/CT (low-dose CT)	3.7 MBq/kg, 130–370 MBq	60 min	Vertex to knee	Visual + semi-quantitative (SUV_max_)	‘FDG accumulation’ above cut-off in ROC
Kaneko et al. [27] ^d,g^	PET/CT (low-dose CT)	3.7 MBq/kg	60 min	Vertex to proximal thigh	Visual + semi-quantitative (SUV_max_) + pattern (diffuse/non-diffuse)	Visual ≥ 2^b^
Lund-Petersen et al. [28] ^g^	PET/CT (low-dose CT)	Unclear	Unclear	Vertex to proximal thigh	Visual	‘Nuclear medicine physician’s description’ based on visual evaluation
Owen et al. [29] ^g^	PET/CT (low-dose CT)	289 ± 30 MBq	60 min	Whole body and dedicated hand images	Visual + semi-quantitative (SUV_max_)	1) Visual ≥ 1^a^ 2) Visual ≥ 2^b^
Owen et al. [30]	PET/CT (low-dose CT)	285 ± 32 MBq (PMR) 276 ± 36 MBq (non-PMR)^†^	60 min	Whole body	Visual + semi-quantitative (SUV_max_)	1) Visual ≥ 1^a^ 2) SUV_max_ cut-off in ROC
Palard-Novello et al. [31] ^g^	PET/CT (low-dose CT)	4 MBq/kg	60 min	Skull base to mid-thigh	Semi-quantitative (SUV_max_)	SUV_max_ > liver
Rehak et al. [33] ^f,g^	PET or PET/CT (low-dose CT or diagnostic/contrast-enhanced CT)	297–483 MBq (median 349 MBq)	55–75 min	Skull base to proximal thigh	Visual + semi-quantitative (SUV_max_) + target-to-liver ratio	Uptake higher than the liver
Rehak et al. [32] ^g^	PET/CT (low-dose CT or diagnostic/contrast-enhanced CT)	327–434 MBq (median 366 MBq)	55–75 min	Skull base to proximal thigh	Semi-quantitative (SUV_max_) + target-to-liver ratio	SUV_max_ > liver
Sondag et al. [34]	PET/CT (low-dose CT)	4.5 MBq/kg	60 min	Vertex to mid-thigh	Visual	1) Visual ≥ 2^b^ 2) Composite PET score cut-off
Takahashi et al. [35]	PET/CT (low-dose CT)	370 MBq	60 min	Vertex to knee	Visual + semi-quantitative (SUV_max_)	1) Visual ≥ 2^b^ 2) Composite PET score cut-off
Wakura et al. [36]	PET/CT (low-dose CT)	185–370 MBq (5–10 mCi)	60 min	Skull to proximal thigh (as suggested by figure and table data)	Visual	1) Visual = 3*^,c^ 2) Composite PET score cut-off
Wendling et al. [37] ^h^	PET/CT (low-dose CT)	4.5 MBq/kg	60 min	Vertex to mid-thigh	Visual	Visual ≥ 1*
Yamashita et al. [39]	PET/CT (low-dose CT)	370 MBq	60 min	vertex to knee	Visual + semi-quantitative (SUV_max_)	1) Visual ≥ 2^b^ 2) Composite PET score cut-off
Yamashita et al. [38]	PET/CT (low-dose CT)	370 MBq	60 min	Vertex to knee	Visual + semi-quantitative (SUV_max_)	Visual ≥ 2^b^
Yuge et al. [40]	PET/CT (low-dose CT)	185 MBq	60 min	Vertex to proximal thigh (as suggested by figure and table data)	Visual + pattern (Y-shaped uptake along the interspinous bursae)	Visual > mediastinal blood pool

*Presumed definition of positive [18F]FDG-PET/CT finding

^†^Mean ± standard deviation

^a^Visual 1 = [18F]FDG uptake less than the liver present

^b^Visual 2 = [18F]FDG uptake equal to the liver present

^c^Visual 3 = [18F]FDG uptake more than the liver present

^d^Study not included in meta-analysis due to lack of relevant data

^e^Study not included in meta-analysis due to inclusion of GCA patients without PMR

^f^Study not included in meta-analysis due to [18F]FDG-PET/CT not performed in every patient (in some patients [18F]FDG-PET scan without CT)

^g^Study not included in meta-analysis due to lack of a control group

^h^Study not included in meta-analysis since it reported [18F]FDG uptake in muscles, which was not reported by other studies

#### Main findings of qualitative assessment

Data regarding the relationship between [18F]FDG-PET/CT and clinical or biochemical findings are provided in Supplementary Table 2. [18F]FDG uptake occurred symmetrically in the shoulder and hip girdles in patients with PMR according to three studies [31, 33, 38]. No convincing relationship was found between [18F]FDG-PET/CT findings and clinical symptoms or inflammatory markers in the blood [21, 23, 28, 31, 37]. One study evaluated the relationship between the age of onset, response to therapy and [18F]FDG-PET/CT findings [22]. This study demonstrated that young PMR patients (age < 60) have a relatively low inflammatory burden on [18F]FDG-PET/CT and poor response to glucocorticoid treatment. Two cross-sectional studies compared [18F]FDG-PET/CT findings between patients with and without concomitant glucocorticoid treatment. Both studies indicated that concomitant glucocorticoid treatment might obscure [18F]FDG-PET/CT findings in patients with PMR [28, 34]. Four studies suggested that [18F]FDG-PET/CT might be useful for monitoring of disease activity in patients treated with glucocorticoids or tocilizumab (anti-IL-6 receptor therapy), as indicated by a reduction of SUV_max_ values and/or the number of positive sites on the scan after initiation of such therapy [24, 31, 32, 35]. Eight studies evaluated large vessel wall uptake; coexisting large vessel vasculitis was observed in 0–40% of patients with PMR [21, 25, 29, 30, 32, 33, 36, 39]. In patients initially suspected of PMR, the [18F]FDG-PET/CT scan identified a malignancy in 3–38% of patients without PMR [25, 26].

### Quantitative analysis (meta-analysis)

#### Studies included in the quantitative analysis

The 9 studies in the meta-analysis reported [18F]FDG-PET/CT findings at distinct anatomic sites rather than an overall positive/negative result of the scan. Two studies reported the diagnostic accuracy of a fixed combination of anatomic sites [21, 30]. Since none of these combinations was reported by more than one study, no meta-analysis was performed for this data. Three unique studies reported the diagnostic accuracy of a composite [18F]FDG-PET/CT score. In case of a potential overlap of patients between studies from the same centre, data from only one study were used according to the criteria listed in the “Methods” section (study selection).

#### Methodological quality of studies in quantitative analysis

**Fig. 2:**
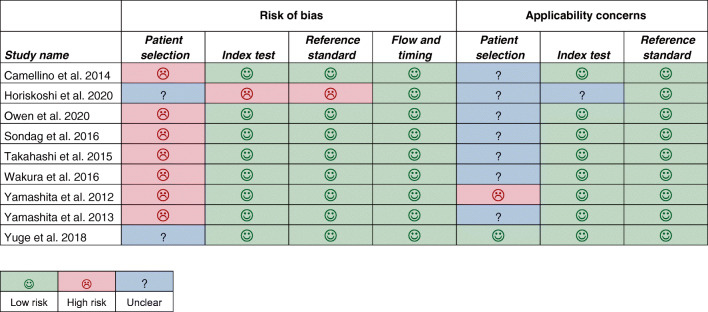
Summary of QUADAS-2 items for the 9 studies included in the meta-analysis

Patient selection was the main source of bias among the 10 studies selected for the meta-analysis (Fig. 2). Two studies did not have a case-control study design and included patients suspected of PMR who underwent a [18F]FDG-PET/CT scan [26, 40]. Even in the latter two studies, it was unclear whether all patients with suspected PMR, or only a selection of those patients, were scanned.

#### Diagnostic accuracy of [18F]FDG-PET/CT for PMR

**Fig. 3:**
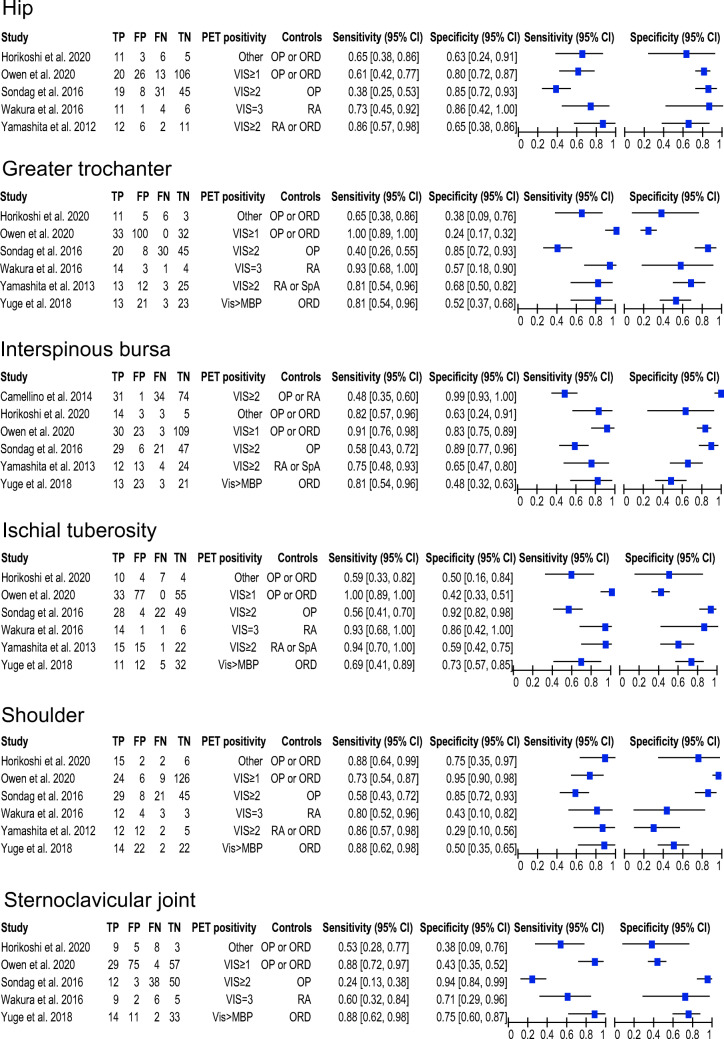
Forest plots showing the sensitivity and specificity of [18F]FDG-PET/CT for PMR. Data are shown for the anatomic sites reported by at least 4 unique studies. VIS visual uptake, OP oncologic patients, ORD patients with other rheumatic disease, RA patients with rheumatoid arthritis, SpA patients with spondyloarthritis, MBP mediastinal blood pool

Table 3 provides an overview of the diagnostic accuracies per anatomic site. The highest pooled sensitivity (> 80%) was observed for positive [18F]FDG uptake at the ischial tuberosity (0.85, 95% CI 0.62–0.95) and greater trochanters (0.83, 95% CI 0.59–0.95), whereas positive [18F]FDG uptake at the interspinous bursae showed the highest specificity (0.81, 95% CI 0.60–0.93). The LR+ was highest for a positive interspinous bursa on [18F]FDG-PET/CT (LR+ 4.00, 95% CI 1.84–8.71), followed by [18F]FDG positive hips (LR+ 2.91; 95% CI 2.09–4.05), ischial tuberosities (LR+ 2.85; 95% CI 1.91–4.25), shoulders (LR+ 2.57; 95% CI 1.24–5.32) and sternoclavicular joints (LR+ 2.31; 95% CI 1.33–4.02). The LR+ for the greater trochanter was not statistically significant. All six anatomic sites yielded relevant negative likelihood ratios of less than 0.5, i.e. ischial tuberosities (LR− 0.21; 95% CI 0.08–0.54), greater trochanters (LR− 0.29; 95% CI 0.13–0.66), interspinous bursae (LR− 0.31; 95% CI 0.21–0.47), shoulders (LR− 0.31; 95% CI 0.19–0.49), hips (LR− 0.47; 95% CI 0.31–0.70) and sternoclavicular joints (LR− 0.49; 95% CI 0.29–0.83). Moderate to high heterogeneity was observed for all anatomic sites as shown in the forest plots and HSROC curves (Fig. 3 and Supplementary Fig. 1). Diagnostic accuracy data regarding sites reported by less than 4 studies are provided in Supplementary Table 3. Three studies reported on a composite [18F]FDG-PET/CT score with a pooled LR+ of 3.91 (95% CI 2.42–6.32) and LR− of 0.19 (95% CI 0.10–0.36) at the optimal cut-off points.

**Table 3:** Diagnostic accuracy of [18F]FDG-PET/CT findings

Site positive on [18F]FDG-PET/CT	No. of patients (no. of cohorts^b^)	Sensitivity (95% CI)	Specificity (95% CI)	Diagnostic OR (95% CI)	LR+ (95% CI)	LR− (95% CI)
Hip	346 (5)	63.7 (46.3–78.1)	78.1 (69.1–85.1)	6.25 (3.32–11.79)	2.91 (2.09–4.05)	0.47 (0.31–0.70)
Greater trochanter	428 (6)	83.3 (59.0–94.5)	56.7 (38.3–73.5)	6.54 (2.87–14.90)	1.93 (1.43–2.59)	0.29 (0.13–0.66)
Interspinous bursa	546 (6)	74.5 (59.3–85.4)	81.4 (59.6–92.8)	12.76 (5.64–28.89)	4.00 (1.84–8.71)	0.31 (0.21–0.47)
Ischial tuberosity	428 (6)	85.4 (62.3–95.4)	70.1 (53.5–82.7)	13.72 (5.20–36.18)	2.86 (1.91–4.28)	0.21 (0.08–0.54)
Shoulder^a^	406 (6)	78.4 (65.4–87.5)	69.5 (42.5–87.5)	8.30 (3.05–22.58)	2.57 (1.24–5.32)	0.31 (0.19–0.49)
Sternoclavicular joint	375 (5)	64.4 (39.1–83.6)	72.1 (48.3–87.8)	4.68 (2.06–10.63)	2.31 (1.33–4.02)	0.49 (0.29–0.83)

Hierarchical logistic regression modelling was used to determine summary estimates of the sensitivity, specificity, diagnostic odds ratio and likelihood ratios by the bivariate model approach. *95% CI* 95% confidence interval, *OR* odds ratio, *LR+* positive likelihood ratio, *LR−* negative likelihood ratio

^a^Data either reported as shoulder or glenohumeral joint

^b^In case of potential data overlap between studies, only data from one study was used according to criteria described in the “Methods” section

## Discussion

### Main findings

This systematic review and meta-analysis summarizes current evidence on the diagnostic value of [18F]FDG-PET/CT for PMR. Estimates of the LRs indicate that shoulders, sternoclavicular joints, interspinous bursae, ischial tuberosities, hips and greater trochanters are important anatomic sites to evaluate in patients with suspected PMR. Concomitant use of glucocorticoid treatment may affect the sensitivity of the [18F]FDG-PET/CT for diagnosing PMR. A limited number of studies suggest that [18F]FDG-PET/CT might be useful for the monitoring of disease activity in patients with PMR. Moderate to high heterogeneity was observed across studies not only due to selection bias, but also due to differences in scanning procedures and interpretation.

Since various articular and extra-articular sites throughout the body can be involved in PMR, a whole-body evaluation of inflammatory activity by [18F]FDG-PET/CT offers significant advantages over localized MRI or ultrasonography [12]. Ultrasonography (sensitivity 66%, specificity 81%) is currently recommended as a diagnostic imaging modality for suspected PMR according to the 2012 provisional ACR/EULAR classification criteria for PMR [4]. Our study indicates that [18F]FDG-PET/CT findings at various individual anatomic sites provide comparable sensitivity and specificity for a diagnosis of PMR. Moreover, composite [18F]FDG-PET/CT scores provided a pooled sensitivity of 85% and a specificity of 80%. Given its higher sensitivity and similar specificity compared to ultrasound, [18F]FDG-PET/CT is a valuable diagnostic tool, especially in patients with clinically suspected PMR and negative ultrasound scan. More recently, combined MRI of shoulders and hips has been shown to allow for a more accurate assessment of joint and peri-articular inflammation compared to ultrasound [10]. Mackie et al. have reported on a typical ‘extracapsular pattern’ on multiple joint MRI, yielding a specificity of 94% and a sensitivity of 64% for diagnosing PMR [7]. Unlike ultrasonography and MRI, [18F]FDG-PET/CT is inherently a whole-body imaging modality and allows evaluating other disorders such as associated large vessel vasculitis or malignancies. Such conditions were indeed identified by [18F]FDG-PET/CT in some of the studies included in our systematic review. Overall, there is accumulating evidence pointing towards a valuable role for [18F]-FDG-PET/CT in the diagnostic work-up of patients with suspected PMR.

Important anatomic sites in the evaluation of suspected PMR by [18F]FDG-PET/CT encompassed the articular and extra-articular structures of the shoulder and pelvic girdle, as well as the spinal column. Although insufficient data precluded evaluation of knee [18F]FDG uptake in the current meta-analysis, it has been suggested that knees can be affected in PMR and should also be evaluated if possible [12, 23, 29, 30]. It would be interesting to know the diagnostic accuracy of fixed combinations of distinct anatomic sites, for instance involvement of shoulders and ischial tuberosities on [18F]FDG-PET/CT. This combination provided a sensitivity of 94% and a specificity of 92% for PMR in one study [30]. However, data for such combinations were too scarce to include in the current meta-analysis. Nevertheless, three unique studies allowed evaluating the diagnostic accuracy of a composite [18F]FDG-PET/CT score for PMR [34–36]. Although the scoring systems were very different, rather homogeneous diagnostic accuracy data were obtained with a pooled sensitivity and specificity of 85% and 80%, respectively. The study by Henckaerts et al., which was omitted from the meta-analysis due to inclusion of patients with [18F]FDG-PET scans, reported a similar diagnostic accuracy for another composite [18F]FDG-PET/CT score [25]. Future studies should determine which composite [18F]FDG-PET/CT score is preferred.

Recently, another meta-analysis by Kim et al. evaluated the diagnostic performance of [18F]FDG-PET/CT for PMR [44]. The latter included two studies that were excluded from our meta-analysis (one due to inclusion of PET scans without CT and the other one because of reporting muscle metabolic activity), whilst 4 additional studies have been included in our meta-analysis [21, 25, 26, 37–39]. Our risk of bias assessment concerning patient selection differed substantially. Most studies in both meta-analyses were case-control studies in which the control subjects were not necessarily suspected of having PMR and were therefore considered to be at high risk for selection bias in our study. The meta-analysis by Kim et al. suggested a pooled sensitivity of 76% and a specificity of 76% of overall [18F]FDG-PET/CT positivity for a diagnosis of PMR, although a precise definition for overall [18F]FDG-PET/CT positivity was not provided. In contrast to the meta-analysis by Kim et al., our study provides more detailed data including the evaluation of composite [18F]FDG-PET/CT scores and diagnostic accuracy of [18F]FDG-PET/CT findings at distinct anatomic sites, as well as an extensive qualitative assessment.

Several factors might have contributed to the between-study heterogeneity observed in the forest plots and HSROC curves. First, differences in methodological aspects of the [18F]FDG-PET/CT scan (e.g. administered activity, scan systems, reconstruction algorithms) could lead to such heterogeneity. Moreover, variation in scoring systems was observed across the included studies. All studies included in the meta-analysis applied a visual uptake scoring system, whilst half of these studies also applied a semi-quantitative parameter (i.e. SUV_max_). The visual grading system mainly used the liver activity as the reference background, but the definition of FDG positivity on a visual scale as well as the optimal SUV cut-off value differed substantially between the studies. This highlights the need for a standardized scoring system for PMR activity on [18F]FDG-PET/CT in addition to standardization of the scanning protocol itself. Importantly, procedural recommendations for [18F]FDG-PET/CT imaging in PMR have recently been reported [12]. The between-study heterogeneity could also be explained by differences in patient characteristics in the included studies. For instance, most studies were case-controlled studies and the selection of the control cohort (e.g. patients with cancer, or rheumatoid arthritis) might have heavily influenced the observed diagnostic accuracy of [18F]FDG-PET/CT.

### Limitations

We do acknowledge further limitations of our study. The number of patients included in the meta-analysis was relatively small. Due to exclusion of non-English reports or conference papers, relevant data may have been omitted. We did not seek to obtain unpublished data via contacting of authors. Various types of bias were present in our study. Most studies had a case-control design. The selection of a control group without symptoms suggestive of PMR (e.g. oncologic patients) might lead to overestimation of the diagnostic accuracy of [18F]FDG-PET/CT for PMR. Additional selection bias may have resulted from the retrospective nature of the majority of the studies. For instance, the decision to perform a [18F]FDG-PET/CT might be based on the clinical suspicion for a malignancy or concomitant large vessel vasculitis. In a minority of studies, some patients had already received glucocorticoid treatment prior to the [18F]FDG-PET/CT, which might have led to underestimation of the diagnostic accuracy. Our systematic review was primarily focused on PMR in the absence of giant cell arteritis, although concomitant vasculitis was observed in part of the included studies. Finally, publication bias is a concern inherent to all meta-analyses. Whilst these factors need to be taken into account, the current study provides the most comprehensive overview of the diagnostic value of [18F]FDG-PET/CT for PMR to date.

## Conclusion

[18F]FDG-PET/CT may be a valuable diagnostic tool in the work-up of patients with suspected PMR, and this study provides insight into specific anatomic sites on [18F]FDG-PET/CT that are informative for a diagnosis of PMR. A composite [18F]FDG-PET/CT score might also be of interest, but agreement on the preferred anatomic sites in such composite score is awaited. Depending on the clinical probability of PMR, [18F]FDG-PET/CT may help to rule in or rule out the diagnosis. Furthermore, [18F]FDG-PET/CT aids in the detection of other serious conditions in part of patients. Further studies are needed to more precisely estimate the diagnostic accuracy of [18F]FDG-PET/CT for PMR. Such studies should ideally have a prospective study design, include all consecutive patients with suspected PMR and adhere to reported procedural recommendations and interpretation criteria for [18F]FDG-PET/CT in PMR.

## Supplementary information

ESM 1 (DOCX 204 kb)
