# Supplementary material for: Diagnostic value of [18F]FDG-PET/CT in polymyalgia rheumatica: a systematic review and meta-analysis
Source: Eur J Nucl Med Mol Imaging. 2020 Dec 28;48(6):1876–89. doi: 10.1007/s00259-020-05162-6 (PMC8113217; doi:10.1007/s00259-020-05162-6)
Supplement: Supplementary file 1 — (DOCX 204 kb) [file 259_2020_5162_MOESM1_ESM.docx]

**Supplementary Figure 1. HSROC plots showing sensitivity and specificity of [18F]FDG-PET/CT for PMR.** Data are shown for the anatomic sites reported by at least four unique studies.

| Hip  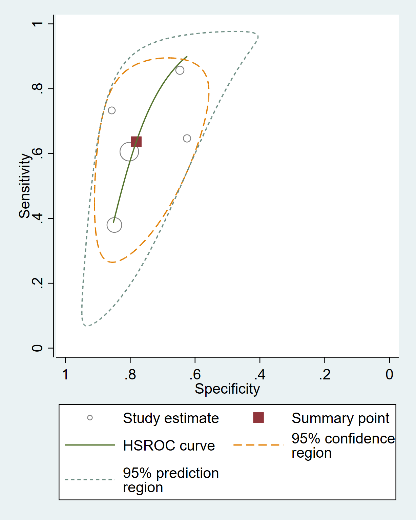 | Greater trochanter  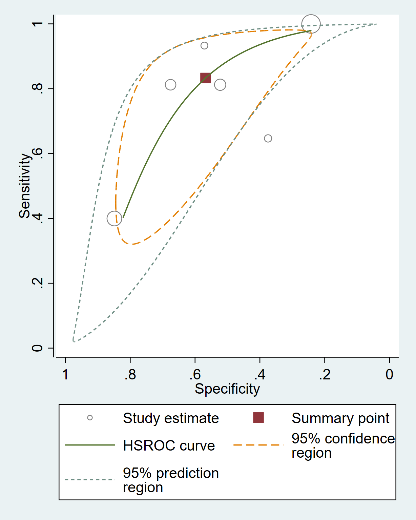 | Interspinous bursa  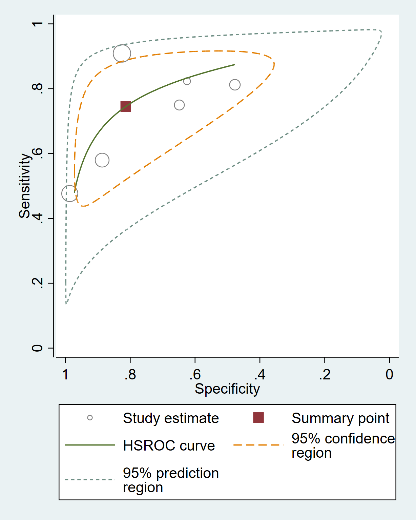 |
| --- | --- | --- |
| Ischial tuberosity  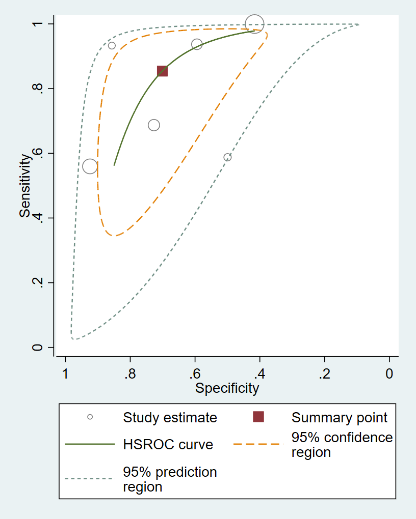 | **Shoulder**  **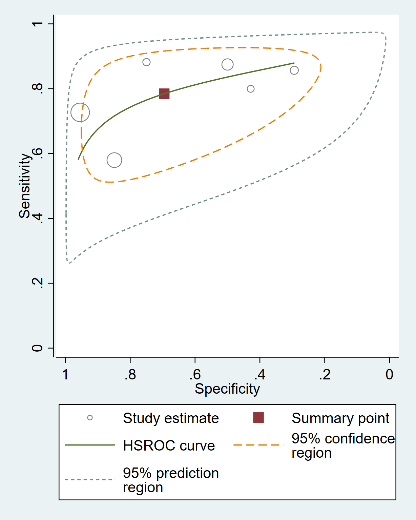** | **Sternoclavicular**  **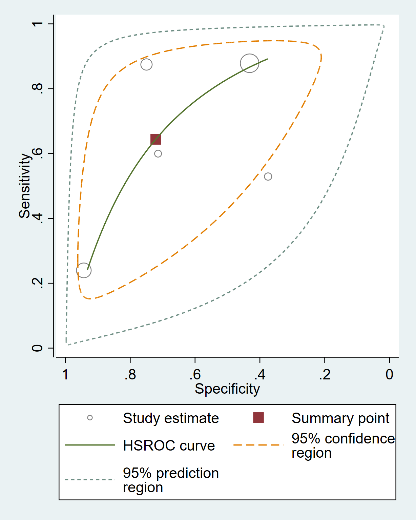** |

**Supplementary Table 1. Composite [18F]FDG-PET/CT scores.** Overview of studies reporting a [18F]FDG-PET/CT score for PMR. Data on the scoring systems are provided.

| **Study** | **Anatomic sites included in the FDG-PET/CT score (total no. of sites)** | **Definition of positive FDG-PET/CT finding** | **Scoring system (range of potential scores)** | **Optimal cut-off reported in study** |
| --- | --- | --- | --- | --- |
| Henckaerts et al. 2018 ^a^ | Cervical spinous processes, lumbar spinous processes, left and right sternoclavicular joint, left and right ischial tuberosity, left and right greater trochanter, left and right hip, and left and right shoulder (n = 12) | No definition used for this FDG-PET/CT score. Visual scoring 0-2 performed (0 = no FDG uptake, 1 = uptake less  than liver uptake, 2 = uptake equal or more than liver uptake) | Sum of visual scores at all sites (Range 0-24 points). | 16 points |
| Sondag et al. 2016 | Two shoulders, two acromioclavicular and two sternoclavicular joints, two greater trochanter, two hips, two ischial tuberosities, two iliopectineal bursitis, two symphysis pubis enthesis and the most inflammatory interspinous bursa (n = 17). | 1) Visual ≥ 2 (FDG uptake equal to/more than liver uptake). | One point per positive site (Range: 0-17 points). | 3 points* |
|  |  | 2) No definition used for this FDG-PET/CT score. Visual scoring 0-3 performed. (0 = no FDG uptake, 1 = uptake less  than liver uptake, 2 = uptake equal to liver uptake, 3 = uptake more than liver uptake) | Mean of all visual scores (Range: 0-3). | Mean score of 0.53* |
| Takahashi et al. 2015 | Ischial tuberosity, spinous process, iliopectinal bursa, absence of uptake at wrists, absence of linear or circular uptake around shoulders (n = 5). | Visual ≥ 2 (FDG uptake equal to/more than liver uptake). | Ischial tuberosity, spinous process, iliopectinal bursa: one point per positive site. Wrists, linear or circular uptake around shoulders: one point if negative (Range 0-5 points). | 3 points † |
| Wakura et al. 2016 | Periarticular area of scapulohumeral  joint, enthesis of pectineus muscle, vicinity of enthesis of rectus femoris muscle,  lateral side of greater trochanter, ischial tuberosity, hip joint, spinous process of lower cervical vertebra, intervertebral joints of lumbar vertebrae, and spinous processes of lumbar vertebrae (n = 9). | FDG uptake more than liver uptake. | One point per positive site  (Range 0-9 points). | 5 points |
| Yamashita et al. 2012 | Ischial tuberosity, greater trochanter, spinous process (n = 3). | Visual ≥ 2 (FDG uptake equal to/more than liver uptake). | One point per positive site  (Range 0-3 points). | 2 points † |

^a^ Study not included in meta-analysis due to FDG-PET/CT not performed in every patient (in some patients FDG-PET scan without CT).

* Data overlap (same study): results of the FDG-PET/CT score of the number of positive sites were used for the meta-analysis, since this scoring systemic encompassed a clear definition of FDG-PET/CT positive sites.

† Data overlap (same hospital, same time period): results of the FDG-PET/CT score of Takahashi et al. were used for the meta-analysis, since this study contained more patients than the study reported by Yamashita et al.

**Supplementary Table 2. [18F]FDG-PET/CT findings and clinical/biochemical aspects of PMR.** NR = not reported.

| **Study** | **FDG-PET/CT abnormalities symmetrical** | **Relationship FDG-PET/CT with age and sex** | **Relationship FDG-PET/CT with symptoms** | **Relationship FDG-PET/CT with inflammation markers** | **Difference between FDG-PET/CT with/without concomitant treatment** | **Detection of large vessel GCA or malignancy on FDG-PET/CT** | **Disease monitoring by FDG-PET/CT** |
| --- | --- | --- | --- | --- | --- | --- | --- |
| Camellino et al.  (2014) | NR | NR | FDG uptake at cervical/lumbar interspinous bursa showed no relationship with local pain or morning stiffness. | FDG uptake at cervical/lumbar interspinous bursa showed no relationship with ESR/CRP. | NR | Patients with PMR included. Carotid artery vasculitis in 5% of patients (not associated with neck pain). | NR |
| Charpentier et al. (2018) | NR | Lower composite FDG-PET/CT score in patients younger than 60 versus those older than 60; young patients were also more frequently male (65%) than old patients (29%). | NR | NR | NR | NR | NR |
| Cimmino et al. (2013) | Complete symmetry of abnormal FDG uptake at various knee structures in 40% of patients. | NR | FDG uptake at knee associated with longer morning stiffness and more pain on movement at pelvic girdle. | FDG uptake at knee showed no relationship with ESR/CRP | NR | NR | NR |
| Devauchelle-Pensec et al. (2016) | NR | NR | NR | NR | NR | NR | Decrease of SUVmax at hips rather than shoulders after 12 weeks of tocilizumab treatment. |
| Henckaerts et al. (2018) | NR | NR | NR | NR | NR | Patients with suspected PMR with/without symptoms of giant cell arteritis included; 15% of PMR patients and 6% of non-PMR patients arterial FDG uptake equal to or more than liver). In 3% of non-PMR patients FDG-PET/CT identified a (potential) malignancy. | NR |
| Horikoshi et al. (2020) | NR | NR | NR | NR | NR | Patients with suspected PMR were included; in 38% of non-PMR patients FDG-PET/CT identified a (potential) malignancy. | NR |
| Kaneko et al. (2020) | NR | NR | NR | NR | NR | NR | NR |
| Lund-Petersen et al. (2017) | NR | NR | FDG uptake at interspinous bursa, hips or ischial tuberosity showed no relationship with local pain. | FDG uptake at individual anatomic sites showed no relationship with CRP. | Completely normal FDG-PET/CT in 33% of patients with PMR taking glucocorticoids; scans were abnormal in all glucocorticoid-naïve patients. | NR | NR |
| Owen et al. (2018) | NR | NR | NR | NR | NR | Patients with PMR were included; no case of large vessel vasculitis on FDG-PET/CT was found. | NR |
| Owen et al. (2020) | NR | NR | NR | NR | NR | Patients with PMR were included; no case of large vessel vasculitis on FDG-PET/CT was found. | NR |
| Palard-Novello et al. (2016) | Complete symmetry of abnormal FDG uptake at sternoclavicular joints, shoulders, hips and ischial tuberosities | NR | NR | Change of overall SUVmax showed no relationship with change of CRP/ESR after 12 weeks of tocilizumab treatment. | NR | NR | Decrease of overall SUVmax and SUVmax at hips, ischial tuberosities and lumbar interspinous bursa after 12 weeks of tocilizumab treatment; trend for decrease of SUVmax at sternoclavicular joints; no decrease of SUVmax at shoulders and cervical interspinous bursa. |
| Rehak et al. (2015) | Complete symmetry of high FDG uptake at shoulders, SC joints, hips, ischiogluteal bursae and praepubic bursae. | NR | NR | NR | NR | Patients with PMR were included; 40% of patients had large vessel GCA on FDG-PET/CT. | NR |
| Rehak et al. (2017) | NR | NR | NR | NR | NR | Patients with PMR were included; 33% of patients had large vessel GCA on FDG-PET/CT. | Decrease of FDG target-to-liver ratio in all evaluated anatomic sites during remission in all patients after treatment with glucocorticoids (median 8 months); nevertheless 7-20% of anatomic sites remained positive. |
| Sondag et al. (2016) | NR | NR | NR | Higher composite FDG-PET/CT scores and more positive anatomic sites in patients with elevated CRP; likely confounded by low CRP values in patients already treated with glucocorticoids. | Higher composite FDG-PET/CT scores and more positive anatomic sites in patients without glucocorticoid treatment versus those with glucocorticoid treatment. | NR | NR |
| Takahashi et al. (2015) | NR | NR | NR | NR | NR | NR | Disappearance of FDG uptake at shoulders, lumbar interspinous bursa and iliopectineal bursae after glucocorticoid treatment is shown for 1 patient. |
| Wakura et al. (2016) | NR | NR | NR | NR | NR | Patients with PMR were included; 7% of patients had large vessel GCA on FDG-PET/CT (in addition to symptoms suggestive of large vessel GCA). | NR |
| Wendling et al. (2020) | NR | FDG uptake at muscles showed no relationship with age. | NR | FDG uptake at muscles showed no relationship with CRP. | NR | NR | NR |
| Yamashita et al. (2012) | Abnormal FDG uptake in both shoulders and hips in 86% of patients. | NR | NR | NR | NR | Patients with PMR were included; 14% of patients had large vessel GCA on FDG-PET/CT (in the absence of symptoms suggestive of large vessel GCA). | NR |
| Yamashita et al. (2013) | NR | NR | NR | NR | NR | NR | NR |
| Yuge et al. (2018) | NR | NR | NR | NR | NR | NR | NR |

**Supplementary Table 3. Diagnostic accuracy of [18F]FDG-PET/CT findings at anatomic sites reported by few studies.** Data are shown for anatomic sites reported by less than four studies. In this case, meta-analysis with the bivariate model could not be performed. Therefore, pooled estimates of diagnostic accuracy parameters were determined with an univariate random-effects model (DerSimonian Laird method). Pooled estimates are only shown if heterogeneity (I^2^) was <75%. 95% CI = 95% confidence interval. OR = odds ratio. LR+ = positive likelihood ratio. LR- = negative likelihood ratio.

| **Site positive on FDG-PET/CT** | **Study** | **Sensitivity**  **(95% CI)** | **Specificity**  **(95% CI)** | **Diagnostic OR**  **(95% CI)** | **LR+**  **(95% CI)** | **LR-**  **(95% CI)** |
| --- | --- | --- | --- | --- | --- | --- |
| Acromioclavicular | Owen et al. 2020 | 100.0 (89.4-100.0) | 33.3 (25.4-42.1) | 33.69 (2.02-562.69) | 1.48 (1.30-1.68) | 0.04 (0.00-0.70) |
|  | Sondag et al. 2016 | 26.0 (14.6-40.3) | 92.5 (81.8-97.9) | 4.30 (1.30-14.28) | 3.45 (1.20-9.86) | 0.80 (0.67-0.96) |
|  | Yuge et al. 2018 | 25.0 (7.3-52.4) | 81.8 (67.3-91.8) | 1.50 (0.38-5.88) | 1.38 (0.48-3.95) | 0.92 (0.67-1.26) |
|  | POOLED | (-) | (-) | 4.22 (0.94-18.97) | 1.76 (0.93-3.35) | (-) |
| Iliopectineal | Sondag et al. 2016 | 31.4 (19.1-45.9) | 92.5 (81.8-97.9) | 5.60 (1.72-18.20) | 4.16 (1.49-11.60) | 0.74 (0.61-0.91) |
|  | Takahashi et al. 2015 | 59.3 (38.8-77.6) | 90.0 (55.5-99.7) | 13.09 (1.45-118.62) | 5.93 (0.90-39.05) | 0.45 (0.28-0.75) |
|  | POOLED | (-) | 92.1 (82.4-97.4) | 6.76 (2.39-19.12) | 4.51 (1.83-11.10) | 0.61 (0.38-0.99) |
| Interspinous cervical | Camellino et al. 2014 | 9.2 (3.5-19.0) | 100.0 (95.2-100.0) | 16.50 (0.91-298.73) | 14.97 (0.86-260.74) | 0.91 (0.84-0.99) |
|  | Wakura et al. 2016 | 46.7 (21.3-73.4) | 100.0 (59.0-100.0) | 13.24 (0.64-272.96) | 7.50 (0.49-115.46) | 0.57 (0.35-0.93) |
|  | POOLED | (-) | 100.0 (95.6-100.0) | 14.85 (1.83-120.35) | 10.44 (1.45-75.24) | (-) |
| Interspinous lumbar | Camellino et al. 2014 | 46.2 (33.7-59.0) | 98.7 (92.8-100.0) | 63.43 (8.31-484.18) | 34.62 (4.85-246.85) | 0.55 (0.44-0.69) |
|  | Wakura et al. 2016 | 73.3 (44.9-92.2) | 85.7 (42.1-99.6) | 16.50 (1.49-183.07) | 5.13 (0.82-32.32) | 0.31 (0.13-0.76) |
|  | POOLED | 51.3 (39.8-62.6) | 97.6 (91.5-99.7) | 36.21 (7.66-171.07) | 12.95 (1.81-92.47) | 0.49 (0.32-0.76) |
| Shoulder peri-articular | Owen et al. 2020 | 97.0 (84.2-99.9) | 18.2 (12.0-25.8) | 7.11 (0.93-54.63) | 1.19 (1.07-1.31) | 0.17 (0.02-1.19) |
|  | Wakura et al. 2016 | 73.3 (44.9-92.2) | 85.7 (42.1-99.6) | 16.50 (1.49-183.07) | 5.13 (0.82-32.32) | 0.31 (0.13-0.76) |
|  | POOLED | (-) | (-) | 10.11 (2.13-47.90) | (-) | 0.28 (0.12-0.63) |
| Wrist | Takahashi et al. 2015 | 40.7 (22.4-61.2) | 0.0 (0.0-30.8) | 0.03 (0.00-0.63) | 0.43 (0.27-0.68) | 12.96 (0.85-197.92) |
|  | Yuge et al. 2018 | 6.3 (0.2-30.2) | 77.3 (62.2-88.5) | 0.23 (0.03-1.93) | 0.28 (0.04-1.98) | 1.21 (0.99-1.49) |
|  | POOLED | (-) | (-) | 0.11 (0.02-0.70) | 0.42 (0.27-0.66) | (-) |
| Composite FDG-PET/CT score | Sondag et al. 2016 | 80.0 (66.3-90.0 | 77.4 (63.8-87.7) | 13.67 (5.31-35.18) | 3.53 (2.11-5.92) | 0.26 (0.15-0.46) |
|  | Takahashi et al. 2015 | 92.6 (75.7-99.1) | 90.0 (55.5-99.7) | 112.50 (9.07-1396.10) | 9.26 (1.44-59.63) | 0.08 (0.02-0.32) |
|  | Wakura et al. 2016 | 86.7 (59.5-98.3) | 85.7 (42.1-99.6) | 39.00 (2.93-518.84) | 6.07 (0.98-37.65) | 0.16 (0.04-0.59) |
|  | POOLED | 84.8 (75.8-91.4) | 80.0 (68.7-88.6) | 24.26 (7.39-79.68) | 3.91 (2.42-6.32) | 0.19 (0.10-0.36) |
